# Towards efficient context-aware classification with compact VLM architectures: indoor fire case study

**DOI:** 10.1038/s41598-026-48743-5

**Published:** 2026-04-14

**Authors:** Anh Tuan GIANG, Nhat Quang DOAN, Minh Duong TRAN, Khanh Dung TRAN, Hoang Ha NGUYEN, Anthony BUSSON

**Affiliations:** 1 University of Science and Technology of Hanoi, Vietnam Academy of Science and Technology, 18 Hoang Quoc Viet, Nghia Do, Hanoi, Vietnam; 2 Hanoi University of Civil Engineering, 55 Giai Phong, Bach Mai, Hanoi, Vietnam; 3 Laboratoire de l’informatique du Parallélisme, UMR 5668, University Lyon 1- ENS de Lyon, UCBL - CNRS - Inria, Lyon, France

**Keywords:** VLM, Context-aware fire classification, LoRA LLM, Classification, Engineering, Mathematics and computing

## Abstract

Accurate and reliable fire detection in indoor environments is critical for ensuring timely emergency responses and enhancing safety in both residential and industrial settings. While recent advances in deep learning have significantly improved fire and smoke detection, most existing systems remain limited to binary classification and often fail to distinguish between hazardous and benign fire events, leading to frequent and disruptive false alarms. To address this limitation, we propose a lightweight and efficient framework for context-aware fire classification. Specifically, input images are first encoded by a visual encoder and then interpreted by a Vision Language Model (VLM) to produce descriptive natural-language captions or semantic embeddings. A language model then processes these representations to perform high-level semantic classification into three categories: no fire, controlled fire (or fire under control), and dangerous fire. This design enables the system to reason about visual context and scene semantics, allowing for nuanced differentiation between visually similar but contextually distinct fire events. Our empirical results, evaluated on both re-labeled public datasets and our custom ConFire dataset, demonstrate that our approach achieves high accuracy while significantly reducing computational overhead. These findings highlight the effectiveness of integrating vision-language reasoning into fire classification tasks, paving the way for next-generation safety monitoring systems that are both intelligent and resource-efficient.

## Introduction

Large Language Models (LLMs) have emerged as transformative tools in artificial intelligence for understanding, generating, and reasoning over natural language. Their generalization ability with zero-shot and few-shot settings has unlocked new possibilities for real-world applications^1^. Building upon the success of LLMs, Visual Language Models (VLMs) extend this capability to multimodal data by combining vision and language understanding^2^. These models can process visual inputs (such as images or videos) and generate corresponding textual representations, or interpret natural-language instructions. By learning joint embeddings of vision and text, VLMs enable applications such as image captioning, visual question answering, and multimodal retrieval^3^. Their ability to reason across modalities makes them particularly appropriate for tasks that require the semantic and contextual interpretation of visual scenes.

Fire detection^4^ is a traditional yet critical application in computer vision, historically addressed through rule-based methods and later through deep learning. CNN-based object detection frameworks, such as YOLO, Faster R-CNN, RetinaNet, and EfficientDet, have been widely adopted for identifying fire and smoke in static images and video streams^5^.

In addition to conventional object detectors, newer multimodal approaches such as BLIP^6^ and CLIP^7^ have been explored for fire and smoke detection. They learns visual concepts by aligning image and text embeddings, enabling it to classify scenes based on natural language prompts. Other techniques include combining image captioning models with NLP-based classifiers to analyze scene descriptions and infer the presence or type of fire. However, a major limitation of existing fire detection systems is their inability to account for context.

In fire detection and classification problems, fires can occur in benign scenarios, such as indoor fireplaces, candles, or controlled industrial flames, where detection without context understanding results in false alarms. These false positives can lead to unwanted situations to fire warnings. Hence, context-aware fire classification is crucial for distinguishing between harmful fire events and those under human control. Complex methods can integrate contextual information to improve classification accuracy^8^.

To address contextual classification, we investigate compact, lightweight VLMs capable of multi-stage image-to-text generation, thereby facilitating contextual classification. Our approach utilizes an image-to-text-to-text pipeline, where a VLM generates descriptive outputs from visual data, which a language model then interprets to classify the situation as ’no fire’, controlled fire’, ’dangerous fire’, or ‘no fire’. This method not only enhances classification accuracy through contextual reasoning but also ensures efficient deployment on low-power, real-time platforms, which are critical for smart surveillance and safety infrastructure.

In this work, we address the challenge of context-aware fire classification and make the following key contributions:

- We collect and annotate a new dataset specifically designed for context-aware fire classification, encompassing three classes: *no fire*, *controlled fire*, and *dangerous fire*.
- We conduct comprehensive experiments on this dataset using state-of-the-art deep learning methods and lightweight VLMs, evaluating their effectiveness for fire understanding.
- We fine-tune the most efficient VLMs to improve the accuracy of context-aware fire classification.

The remainder of this report is organized as follows. Section “Related work” reviews existing literature on fire classification and detection. In Section “Methodology”, we present our lightweight image-to-text-to-text pipeline and the preparation of our dataset. Section "Results and discussion" discusses the experimental results of various VLMs across different datasets and explains the fine-tuning strategies employed. Finally, Section “Conclusion” concludes the report and outlines potential directions for future research.

## Related work

Early research on fire and smoke detection primarily relied on handcrafted features and traditional image processing techniques. Notably^9^, proposed that combining color-based rules with probabilistic modeling (GMM) can provide high fire detection accuracy in both indoor and outdoor scenarios.

The advent of deep learning revolutionized fire detection by enabling automatic feature learning through convolutional neural networks (CNNs). Several works also used deeper architectures, such as ResNet and EfficientNet^10^, for fire classification, achieving higher accuracy on specialized datasets^11^. evaluated several temporal and hybrid deep learning models, including LSTM, Bi-LSTM, GRU, LSTM-FCN, InceptionTime, and Transformer, to detect and classify fire events. Furthermore, YOLO^12^ has been widely adopted for fire and smoke detection in both indoor and outdoor environments. Nevertheless, most of these models perform binary classification, simply identifying the presence or absence of fire, without accounting for context, such as whether the fire is under control or potentially hazardous.

To address the limitations of binary fire detection, recent efforts have focused on developing context-aware classification methods. These approaches integrate scene semantics, object relations, and environmental cues to differentiate fire types^13^. In^14^, the authors introduced FIRE-TASTIC, a novel framework for fire detection in video streams that combines task-aware object detectors with zero-shot vision-language models (VLMs) for enhanced robustness and generalization. FIRE-TASTIC can spot even subtle fire cues and use the semantic reasoning capabilities of VLMs and visual question answering (VQA) to reduce false positives and interpret complex visual contexts.

Recent research has begun to leverage VLMs for understanding fire scenes through multimodal reasoning. This approach integrates vision and language into a unified framework, enabling both image captioning and prompt-based classification. FireCLIP, presented in^15^, proposes a novel fire-detection framework that uses Vision-Language Models (VLMs) with multimodal prompt tuning to address forest fire detection.

Many existing studies, including FIRE-TASTIC and FireCLIP, focus on binary classification to determine whether a scene contains fire. In contrast, our work is concerned with identifying extended fire-related context across diverse indoor scenarios.

## Methodology

This section presents the methodological framework developed for context-aware indoor fire classification using VLMs. Unlike conventional fire detection approaches that treat fire recognition as a simple binary problem, our framework explicitly considers the surrounding scene context to distinguish between dangerous fire, controlled fire, and no fire situations. To support this task, we first introduce two datasets, including ConFire, a newly collected context-aware indoor fire dataset, and a re-annotated version of FireNet adapted to the same three-class setting. We then describe a zero-shot structured prompting pipeline in which VLMs first generate a semantic description of the scene and subsequently infer the fire category based on both visual evidence and contextual reasoning. Finally, we present the evaluation metrics used to assess classification performance.

### Datasets for indoor context-aware fire classification

#### ConFire - context-aware fire dataset

In conventional fire detection systems, especially those based on classical computer vision or early fuzzy logic techniques, classification is often limited to a binary decision: fire or no fire. While this binary model is computationally efficient and easy to implement, it cannot handle complex scenarios in which fire may be present but fully under control (e.g., a campfire, fireplace, or stove). As a result, such systems are prone to generating false alarms, especially in urban and indoor environments where benign fire occurrences are common. For this purpose, we collect indoor images from diverse sources to build a context-aware dataset for indoor fire classification.

**Fig. 1:**
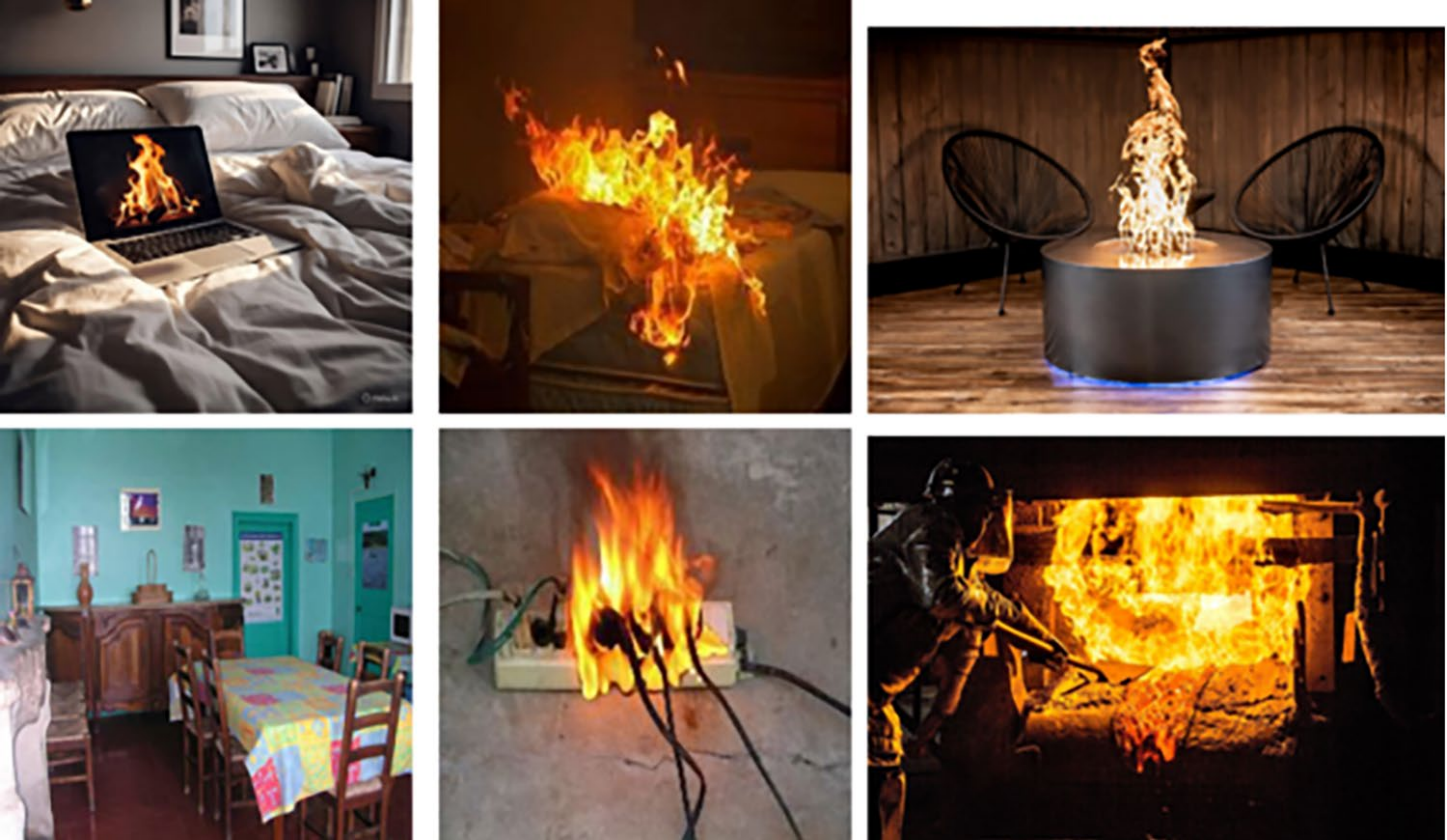
Examples from the dataset: (left) no fire, (middle) dangerous fire, (bottom) controlled fire.

In this dataset, fire or flame can be found in different scenarios:

- Dangerous fire: curtains on fire, smoke on the ceiling, couch on fire, bed sheet on fire, spreading fire towards flammable furniture, etc.
- Controlled fire: active fireplace, campfire, cooking, candles, lighter, etc.
- No fire: fire alarm, fire extinguisher, laptop displaying fire, painting or drawing of fire, etc.

The dataset is available in the link https://www.kaggle.com/datasets/duongtm3012/confire. Table 1 shows the data summarization. The dataset includes three distinct categories: dangerous fire, controlled fire, and no fire. We use Qwen2.5-7B https://huggingface.co/Qwen/Qwen2.5-7B-Instruct to classify and label the images. These labels reflect real-world variability and support the development of smarter, semantically aware fire detection systems. Sample images of each category are illustrated in Fig. 1.

**Table 1:** Number of images per class in the fire classification dataset.

Dataset	Class	# of Images	Total
ConFire	Controlled fire	50	452
	Dangerous fire	83	
	No fire	319	
FireNet	Controlled fire	179	503
	Dangerous fire	321	
	No fire	2	

#### FireNet dataset

The FireNet dataset, available at https://github.com/OlafenwaMoses/FireNET, provides a valuable resource for fire detection tasks. In this work, we re-annotate the images from this dataset to align with the requirements of context-aware fire classification, introducing labels that distinguish between ‘no fire’, ‘controlled fire’, and ‘dangerous fire’. Table 1 shows the summary of this dataset.

**Fig. 2:**
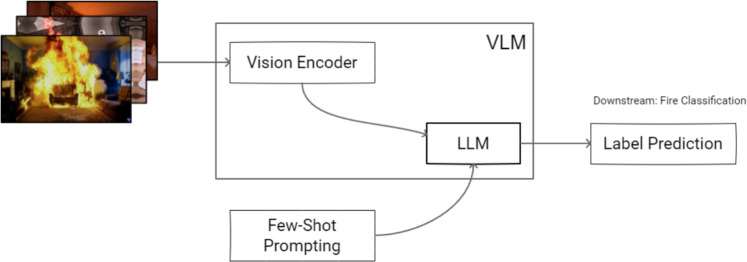
Context-aware classification-based approach using VLMs.

### VLMs with zero-shot structured prompting

We propose a pipeline as in Fig. 2. For the VLM-based methods, we use the entire dataset for testing via a zero-shot prompting approach. This approach is not trained on the dataset but instead relies on pre-trained multimodal understanding to interpret both visual and contextual cues. The process starts with input images that are analyzed by a Vision Encoder (VE) to extract rich visual representations. These visual features are then fed into a large language model (LLM), which is further guided by zero-shot prompting, providing task-relevant examples or instructions to steer the model’s response. An example of the prompt used for inference is shown below:
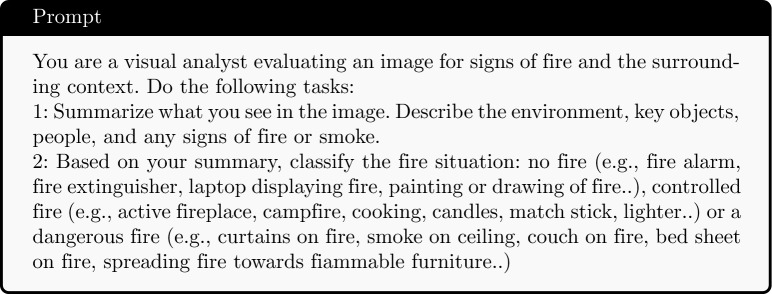


This pipeline follows an image-to-caption-to-label strategy, implementing caption-guided classification via prompt-based reasoning, where the model first generates a natural language description of the scene and then performs classification. This is not only more interpretable but also aligns with the autoregressive nature of large language models. Since LLMs predict tokens sequentially, each token is generated based on the preceding ones. By prompting the model first to produce a descriptive caption of the input image, we provide a coherent semantic context for the classification decision that follows. This ordering encourages more accurate reasoning, as the classification step benefits from the structured, grounded context already established in the caption.

### Quality measures

To evaluate the performance of the proposed fire classification models, we adopt a set of conventional quality metrics commonly used in classification tasks: accuracy, precision, recall, and F1-score. Accuracy provides an overall measure of correctness by capturing the proportion of correctly classified samples. Precision quantifies the model’s ability to avoid false positives, which is particularly important in applications like fire detection, where incorrect alerts may lead to unnecessary responses. Recall measures the model’s sensitivity towd true positive cases, ensuring that genuine fire events are not overlooked. Finally, the F1-score balances precision and recall, offering a single metric that reflects both correctness and completeness. Together, these metrics provide a comprehensive evaluation of each model’s classification capability within the context-aware fire detection framework.

**Table 2:** Comparison of methods on the ConFire dataset. VLM results are reported using the entire ConFire dataset (100%), while DL-based methods are evaluated on the 20% test split of ConFire.

Method	Precision	Recall	F1-Score	CPU	GPU
				(s/img)	(s/img)
EfficientNet$^*$	0.867	0.829	0.783	-	-
ResNet50$^*$	0.914	0.903	0.911	-	-
Qwen2-2B-Instruct$^1$	0.927	0.876	0.892	8.604	2.021
**Qwen2.5-3B-Instruct**$^2$	**0.945**	**0.941**	**0.942**	12.368	3.504
Qwen2.5-3B-F16$^3$	0.745	0.811	0.776	10.251	2.197
pixtral-12B-Q2$^4$	0.857	0.755	0.800	14.650	2.892
Gemma-3-4b-F16$^5$	0.844	0.762	0.799	13.205	3.385
InternVL3-1B-Instruct$^6$	0.886	0.878	0.881	5.482	0.276
InternVL3-1B-F16$^7$	0.684	0.706	0.692	**2.468**	**0.836**
**InternVL3-2B-Instruct**$^8$	**0.953**	**0.930**	**0.915**	6.030	0.520
InternVL3-2B-F16$^9$	0.781	0.861	0.819	5.264	1.046

$^1$https://huggingface.co/ggml-org/Qwen2-VL-2B-Instruct-GGUF.

$^2$https://huggingface.co/Qwen/Qwen2.5-VL-3B-Instruct.

$^3$https://huggingface.co/unsloth/Qwen2.5-VL-3B-Instruct-GGUF.

$^4$https://huggingface.co/mradermacher/pixtral-12b-GGUF.

$^5$https://huggingface.co/unsloth/gemma-3-4b-it-GGUF.

$^6$https://huggingface.co/OpenGVLab/InternVL3-1B-Instruct.

$^7$https://huggingface.co/unsloth/InternVL3-1B-GGUF.

$^8$https://huggingface.co/OpenGVLab/InternVL3-2B-Instruct.

$^9$https://huggingface.co/unsloth/InternVL3-2B-Instruct-GGUF.

## Results and discussion

This section presents and discusses the experimental results of the proposed context-aware fire classification framework. We first evaluate both CNN-based baselines and lightweight VLMs on ConFire. We then analyze the impact of LoRA-based fine-tuning under different adaptation settings and hyperparameter configurations. Finally, we assess the cross-dataset generalization of the selected VLMs on the FireNet benchmark, highlighting the effects of fine-tuning and class-weighted training.

### Evaluation on ConFire

The ConFire dataset is used to evaluate the performance of various methods, including both CNN-based and VLM models, on the task of context-aware fire classification. The CNN-based methods are trained using 80% of the images over 10 epochs, with the remaining 20% reserved for testing. The pre-trained DL-based models serve as supervised baseline methods to provide a reference for evaluating the performance on the dataset. Our main goal is to assess their performance and suitability for distinguishing between different fire scenarios, such as dangerous fires, controlled fires, and non-fire scenes. In addition, we select lightweight VLMs with approximately 1B to 3B parameters to study their reasoning capabilities.

Table 2 presents a comparative analysis of these models on the task of context-aware fire classification. In addition to the quality metrics, we also measured the average running time per image for the VLMs running on an Intel(R) Xeon(R) Gold 6133 processor at 2.50GHz and an NVIDIA GeForce GTX 3090 GPU.

#### CNN baseline results

**Fig. 3:**
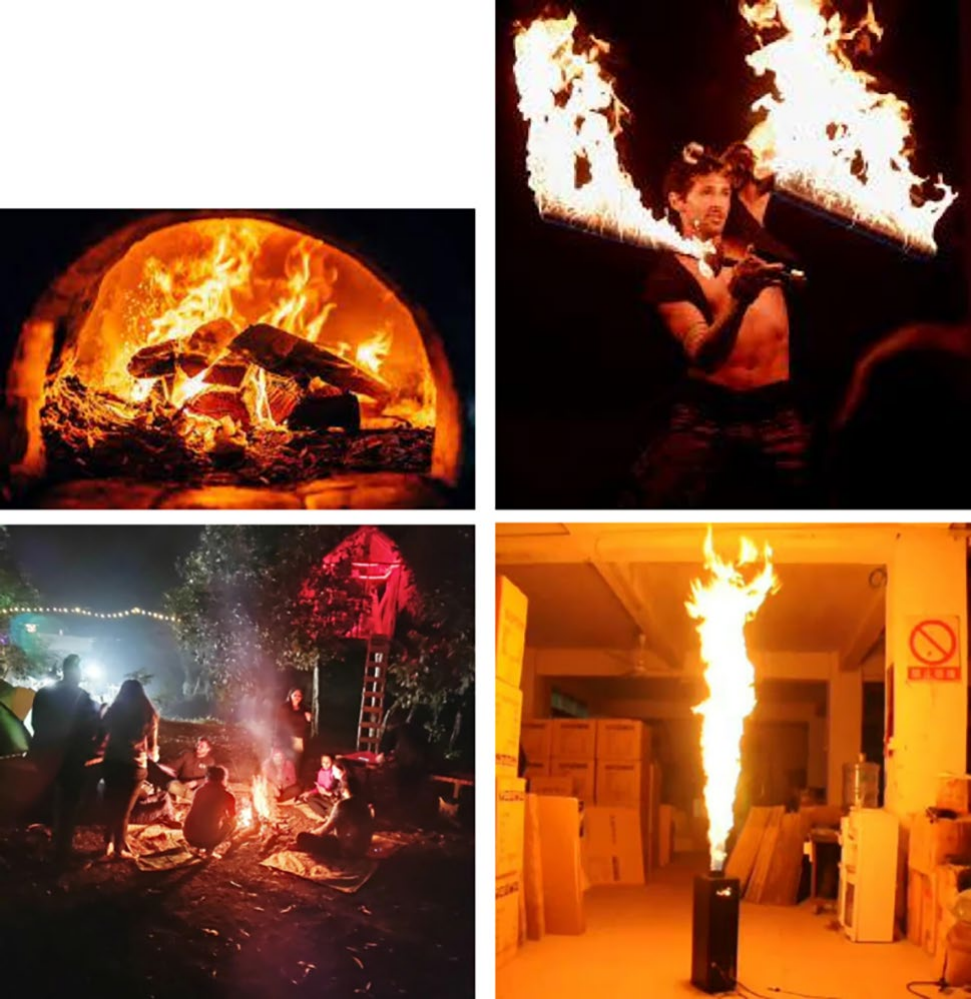
Examples misclassified by ResNet50 on ConFire: ground-truth: ’controlled fire’; prediction: ‘dangerous fire’.

Traditional pre-trained CNN models, such as ResNet50 and EfficientNet, are used as baseline methods for comparison with the VLM approaches. Both models are initialized with pretrained weights and fine-tuned on the training portion of the ConFire dataset. Although they demonstrate reasonable baseline performance, both EfficientNet and ResNet50 exhibit cases in which controlled fire instances are misclassified as dangerous fires. Representative examples of such misclassifications are illustrated in Fig. 3. This behavior indicates a limitation of CNN-based methods in capturing contextual information, which is crucial for correctly interpreting fire-related situations.

#### VLM results

Table 2 shows that multi-modal VLMs significantly outperform CNNs in context-sensitive scenarios. Notably, Qwen2.5-VL-3B-Instruct^16^ achieves the highest scores across all metrics: accuracy (0.941), precision (0.945), recall (0.941), and F1-score (0.942). Comparable results are observed with InternVL3-2B-Instruct. This suggests that incorporating language-guided reasoning into the classification pipeline enhances the model’s understanding of nuanced differences, such as ‘controlled fire’ versus ‘dangerous fire’.

From the efficiency perspective, smaller VLMs such as InternVL3-1B-Instruct and InternVL3-2B-Instruct achieve competitive performance while maintaining relatively low inference latency, particularly on GPU. This indicates that lightweight VLMs can provide a favorable balance between classification accuracy and computational efficiency.

#### Error analysis

**Table 3:** Confusion matrix for Qwen2.5-VL-3B. Rows indicate ground-truth labels and columns indicate predicted labels.

True label $\backslash$ Predicted label	No fire	Controlled fire	Dangerous fire
No fire	311	8	0
Controlled fire	1	49	0
Dangerous fire	4	18	61

**Fig. 4:**
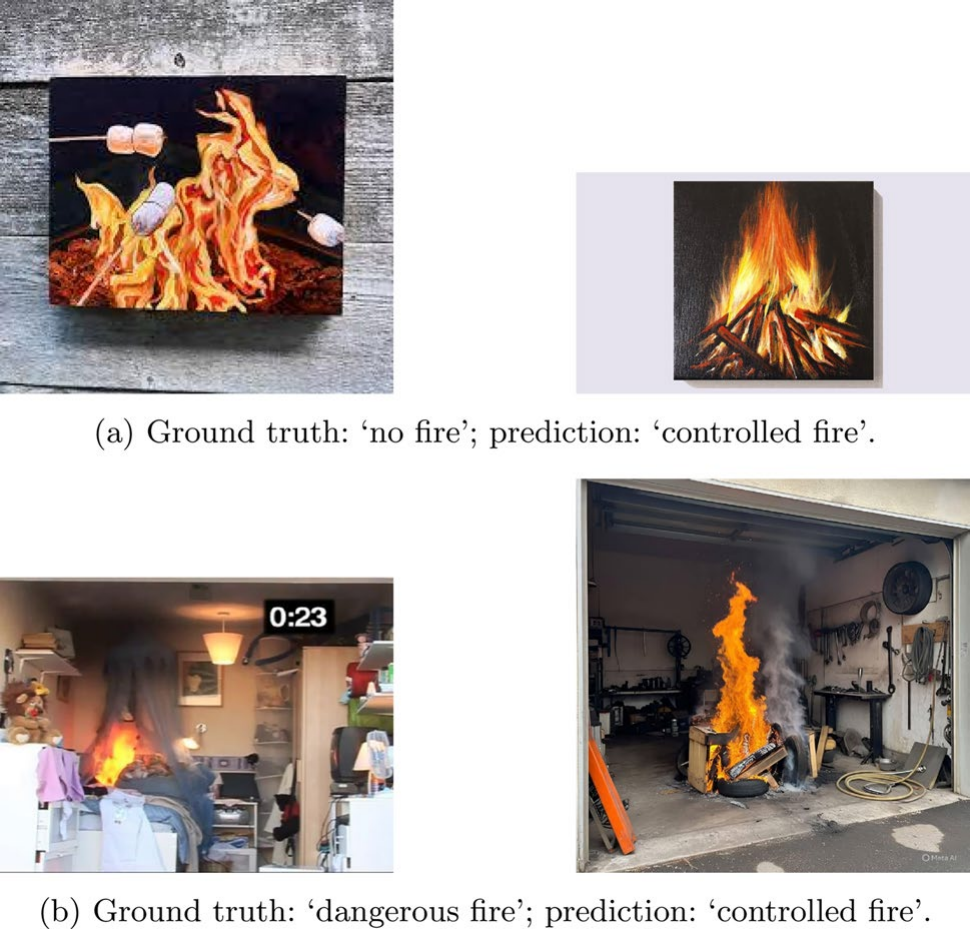
Examples misclassified by Qwen2.5-VL-3B on ConFire.

Among the selected VLMs, Qwen2.5-VL-3B and InternVL3-2B-Instruct achieve the best overall performance. To further understand the behavior of these models, we analyze representative misclassification cases.

Table 3 presents the confusion matrix for Qwen2.5-VL-3B, showing that most errors occur between the ‘controlled fire’ and ‘dangerous fire’ categories. In particular, several ‘dangerous fire’ instances are misclassified as ‘controlled fire’, suggesting that the model sometimes struggles to distinguish hazardous fire events from visually similar but less severe scenarios.

Figure 4 illustrates representative failure cases of Qwen2.5-VL-3B. In Fig. a, images with the ground-truth label ‘no fire’ are misclassified as ‘controlled fire’, indicating that the model may over-rely on visible flame-like patterns or fire-related visual cues. In Fig. b, images labeled as ‘dangerous fire’ are also predicted as ‘controlled fire’, further highlighting the challenge of differentiating between controlled and dangerous fire situations.

### LoRA fine-tuning of VLM models

Based on the previous experiment, we select and fine-tune the Qwen2.5-VL-3B-Instruct and InternVL3-2B-Instruct models using the ConFire dataset, with the FireNet dataset serving as an external evaluation benchmark.

#### Fine-tuning settings

**Fig. 5:**
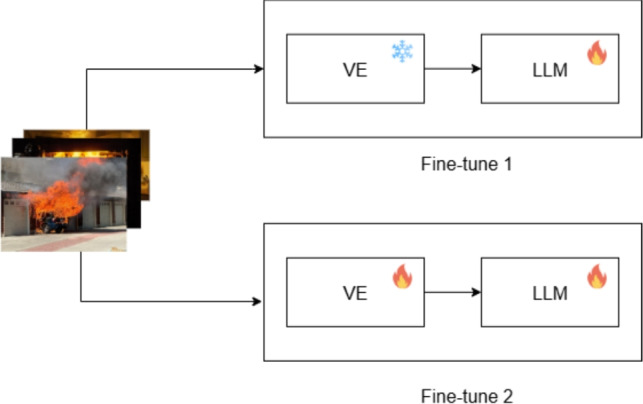
Fine-tune VLMs with two different scenarios.

In the architecture of a Visual Language Model (VLM), two main components are involved: the Large Language Model (LLM) and the Visual Encoder (VE). We explore two fine-tuning strategies as shown in Fig. 5:

- **Fine-Tune 1 (FT1)**: Only the LLM is trainable, while the VE is kept frozen.
- **Fine-Tune 2 (FT2)**: Both the LLM and VE are set as trainable components.

#### Training setup and hyperparameters

To ensure reproducibility and fair comparison between the two experiments, we fixed the following hyperparameters when fine-tuning our VLMs. LoRA adapters were applied to all query/key/value matrices in the LLM layers, while the vision encoder remained frozen for FT1 and trainable for FT2.

**Table 4:** Hyperparameter Settings for VLM Fine-tuning.

Hyperparameter	Value
Adapter method	LoRA
LoRA rank	8
LoRA alpha	16
LoRA dropout	0.05
Loss function	Cross-entropy
Optimizer	AdamW
Initial learning rate	$2\times 10^{-4}$
LR scheduler	Cosine-annealing with linear warmup
Warmup ratio	3 % of total steps
Number of epochs	1
Batch size	2
Gradient clipping	0.3
Precision	bfloat16
Quantization format	4-bit uniform

- We use the standard cross-entropy loss
  $$\begin{aligned} \mathcal {L} = -\sum _{c} y_c \,\log (\hat{y}_c), \end{aligned}$$
  where $y_c$ is the ground-truth one-hot label and $\hat{y}_c$ the predicted probability for class $c$. This loss encourages the model to assign high probability to the correct tokens while penalizing incorrect tokens.
- Trainable low-rank matrices (rank = 8, scaling factor $\alpha$ = 16 and dropout = 0.05) are injected into each attention block of the LLM to help regularize these adapters, reducing the total number of trainable parameters by over 90 %.
- We use AdamW for decoupled weight decay, with an initial learning rate of $2\times 10^{-4}$. A cosine-annealing schedule is applied, with a 3 % linear warmup to stabilize early updates.
- A micro-batch size of 2 in bfloat16 (mixed precision) provides a good trade-off between GPU memory usage and numerical stability on the RTX 3090s.
- We clip gradients at 0.3 to avoid exploding gradients during fine-tuning with a small batch size.
- All models are quantized to 4-bit precision, reducing memory footprint and accelerating inference with minimal impact on accuracy.

**Fig. 6:**
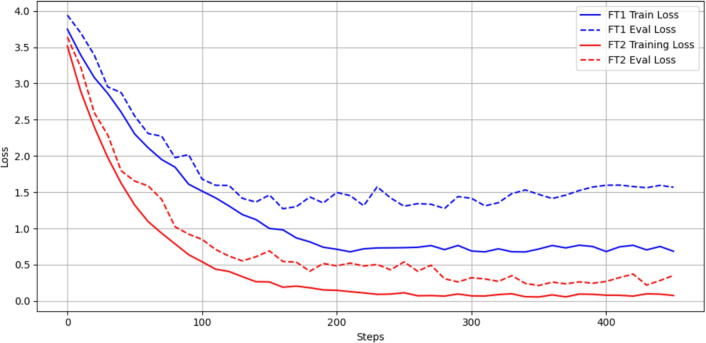
Training and evaluation cross-entropy loss curves of the fine-tuned Qwen2.5-VL models on the ConFire dataset (training) and FireNet dataset (evaluation), respectively.

The training and evaluation loss curves for FT1 and FT2 for Qwen2.5-VL are illustrated in Fig. 6, providing insight into their convergence behavior and generalization performance. Based on the results, FT2 should be better for fine-tuning VLMs. Therefore, we adopt the FT2 approach, where the vision encoder (VE) is unfrozen and both the VE and the LLM are fine-tuned to improve performance on the FireNet dataset.

#### Hyperparameter sensitivity analysis

The objective of performing a hyperparameter sweep is to systematically evaluate how different hyperparameter settings affect the model’s performance and training behavior. To isolate the effect of each parameter, we keep all other parameters fixed and conduct two additional experiments to examine the impact of LoRA hyperparameters. Specifically, Experiment 1 increases the LoRA dropout to 0.1, while Experiment 2 increases the LoRA alpha to 32. The results are compared with the baseline configuration (dropout = 0.05, alpha = 16).

**Fig. 7:**
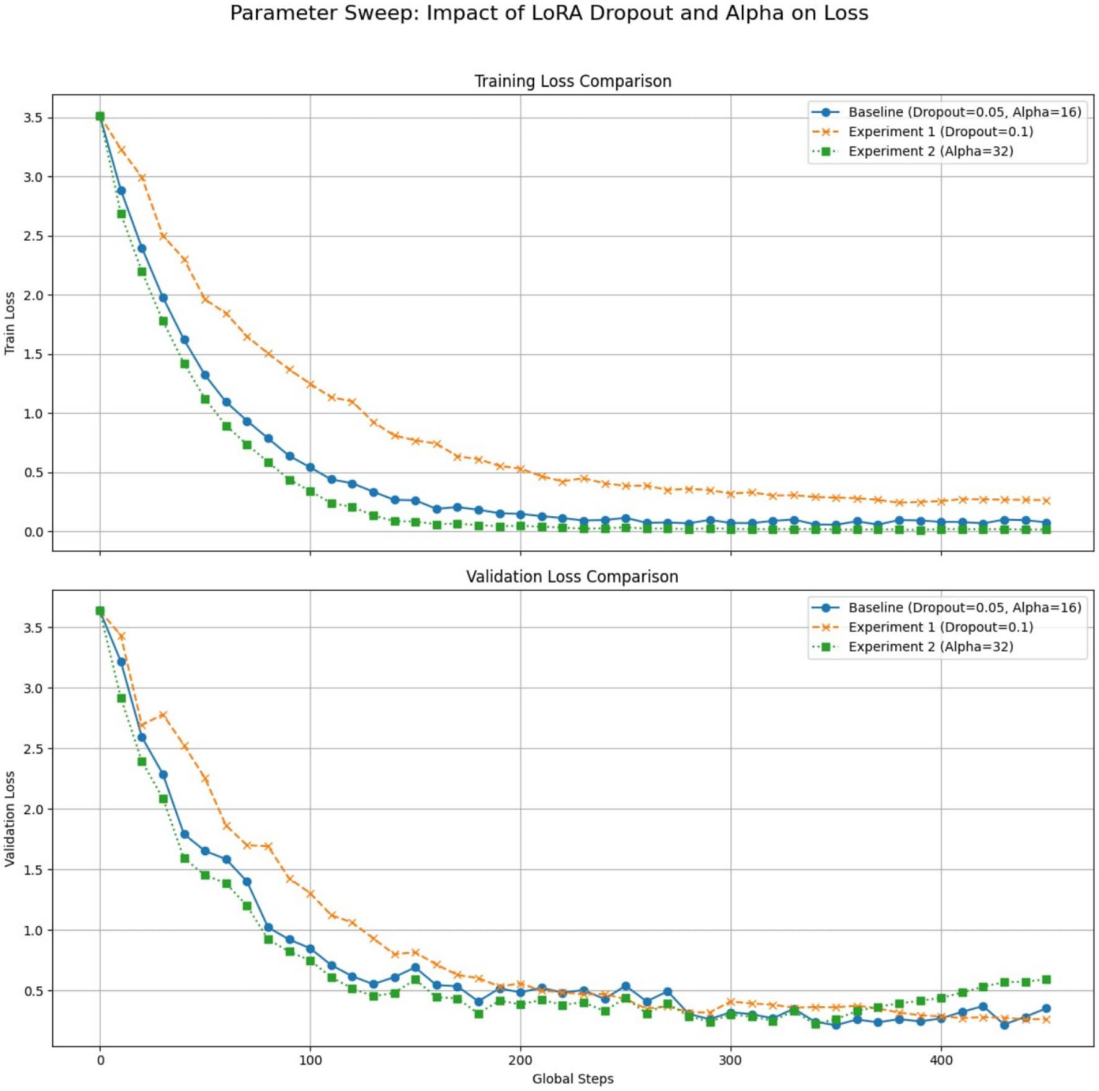
Hyperparameter sweep: impact of LoRA dropout and alpha on training and evaluation loss.

Figure 7 illustrates the training and evaluation loss curves for these experiments. In Experiment 1, increasing the dropout results in a slightly slower but smoother decrease in training loss compared to the baseline, while the evaluation loss eventually reaches a lower value, indicating improved generalization. In Experiment 2, increasing the LoRA alpha to 32 accelerates the reduction of training loss; however, the evaluation loss increases toward the end of training, suggesting potential overfitting.

### Cross-dataset evaluation on FireNet

To evaluate the impact of fine-tuning, we conduct three sets of experiments on two selected VLMs: (1) evaluation before fine-tuning, (2) evaluation after fine-tuning using the ConFire dataset, and (3) evaluation after fine-tuning with class-weighted loss to address class imbalance. The corresponding results are reported in Table 5.

**Table 5:** Fine-tuned VLMs (LLM and VE) on FireNet.

Method	Average	Precision	Recall	F1-Score
Before fine-tuning				
Qwen2.5-VL-3B-Instruct	Micro	0.842	0.842	0.842
	Macro	0.641	0.545	0.588
InternVL3-2B-Instruct	Micro	0.681	0.681	0.681
	Macro	0.634	0.372	0.388
After fine-tuning				
Qwen2.5-VL-3B-Instruct	Micro	0.976	0.976	0.976
	Macro	0.786	0.981	0.841
InternVL3-2B-Instruct	Micro	0.806	0.806	0.806
	Macro	0.672	0.819	0.555
After fine-tuning with class weights				
Qwen2.5-VL-3B-Instruct	Micro	0.986	0.986	0.986
	Macro	0.793	0.989	0.848
InternVL3-2B-Instruct	Micro	0.837	0.837	0.837
	Macro	0.672	0.847	0.582

Table 5 compares the performance of Qwen2.5-VL-3B-Instruct and InternVL3-2B-Instruct on the FireNet dataset before and after fine-tuning. Before fine-tuning, Qwen2.5-VL-3B-Instruct performs better, achieving a micro F1-score of 0.842, while InternVL3-2B-Instruct reaches 0.681. The lower macro scores for both models suggest difficulty in predicting minority classes. After fine-tuning, both models improve. Qwen2.5-VL-3B-Instruct reaches a micro F1-score of 0.976, while InternVL3-2B-Instruct improves to 0.806.

Using class weights during fine-tuning further improves performance. Qwen2.5-VL-3B-Instruct achieves the best results with a micro F1-score of 0.986, while InternVL3-2B-Instruct increases to 0.837. Overall, fine-tuning significantly improves both models, and class-weighted training provides additional gains, with Qwen2.5-VL-3B-Instruct consistently outperforming InternVL3-2B-Instruct.

**Table 6:** Confusion matrix for Qwen2.5-VL-3B fine-tuned and tested on FireNet.

True label $\backslash$ Predicted label	Controlled fire	Dangerous fire	No fire
Controlled fire	170	4	3
Dangerous fire	5	318	0
No fire	0	0	2

### Ablation study

In the ablation study, we compare the proposed framework with two alternative approaches: direct classification and caption-guided classification with minimal guidance. Direct classification predicts the label directly from the image without intermediate reasoning. Caption-guided classification first generates a description of the scene and then outputs the label within the same response. In contrast, the proposed framework introduces a structured reasoning process that explicitly analyzes contextual elements before performing classification, enabling a more reliable interpretation of fire-related scenarios. This study helps validate the effectiveness of the proposed approach.

#### Direct classification

In this study, the VLM receives the input image and is prompted to predict the fire category directly without generating any intermediate description. The model produces the final label (e.g., no fire, controlled fire, or dangerous fire) based solely on its visual understanding and pretrained multimodal knowledge. This setting serves as a baseline to evaluate the model’s ability to perform image-to-label classification without explicit reasoning guidance. The prompt is as follows for all VLMs:
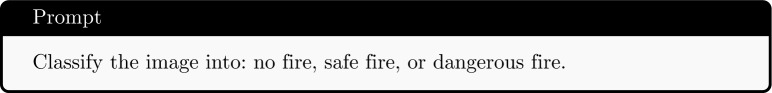


#### Caption-guided classification

In the caption-guided classification with minimal guidance setting, the VLM is prompted to first describe the image’s visual content and then determine the fire category based on the generated description. Both steps are executed within a single VLM response, where the intermediate description helps the model capture contextual cues such as environment, objects, and fire behavior before producing the final label. This approach encourages structured reasoning and can improve classification accuracy in visually ambiguous scenarios. The prompt is as follows for all VLMs:
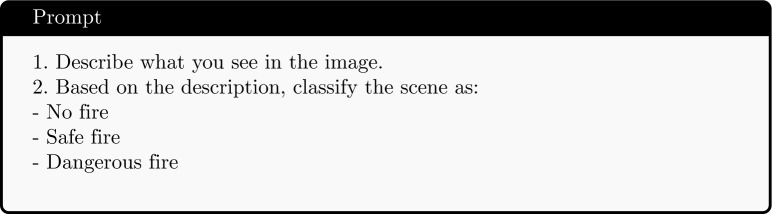


#### Result discussion

Table 7 shows that direct image classification is more effective and efficient than caption-guided classification on ConFire. InternVL3-2B achieves the best direct-classification performance, with a micro F1-score of 0.882, while Qwen2.5-VL-3B obtains competitive results at 0.854. In contrast, caption-guided classification incurs substantially higher inference cost and lower macro-level performance for both models, with a particularly severe degradation for InternVL3-2B. The caption-guided setting degrades sharply to a micro F1 of 0.449, indicating that the generated captions likely fail to preserve enough discriminative information for reliable downstream classification.

Compared with the results in Table 2, all VLM results in Table 7 show inferior performance. While direct classification is generally more competitive than caption-guided classification, both strategies fall short of our method. This confirms the benefit of the proposed framework for accurate and efficient ConFire classification.

The results suggest that general-purpose prompting alone is insufficient for reliable prediction. Incorporating both the image and its textual description can improve classification performance by providing complementary visual and semantic cues. Moreover, carefully designed prompts can further enhance performance, especially for contextual fire classification where subtle scene understanding is required.

**Table 7:** Ablation study of classification strategy on ConFire.

VLM	Average	Precision	Recall	F1-score	Time (sec$/$image)
Direct classification					
Qwen2.5-VL-3B	Micro	0.854	0.854	0.854	1.05 ± 0.15
	Macro	0.861	0.700	0.753	
InternVL3-2B	Micro	0.882	0.882	0.882	0.91 ± 0.30
	Macro	0.819	0.770	0.780	
Caption-guided classifications					
Qwen2.5-VL-3B	Micro	0.865	0.865	0.865	7.84 ± 2.21
	Macro	0.739	0.661	0.679	
InternVL3-2B	Micro	0.449	0.449	0.449	5.52 ± 1.50
	Macro	0.379	0.354	0.321	

**Fig. 8:**
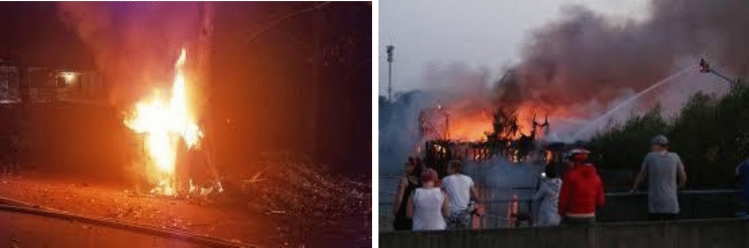
Ground truth: ‘dangerous fire’; prediction: ‘controlled fire.’.

Fig. 8 presents two misclassified samples by Qwen2.5-VL-3B after fine-tuning. In the first image, the model incorrectly predicts the scene as a campfire. In the second image, the presence of several people surrounding the fire also leads the model to interpret the scene as a campfire. These examples suggest that the model may rely heavily on contextual cues such as human activity around the fire, which can sometimes lead to incorrect classification. These cases indicate that performance could be further improved by expanding the dataset and refining the prompt design to better capture contextual information in challenging fire scenarios.

## Conclusion

In this work, we introduce a context-aware fire classification framework that leverages lightweight Visual Language Models. We curated and re-annotated a fire image dataset to support fine-grained classification into three scenarios: no fire, controlled fire, and dangerous fire. Through extensive experiments, we evaluated both conventional deep learning models (e.g., ResNet50, EfficientNet) and state-of-the-art VLMs (e.g., Qwen2.5-VL-3B, InternVL3-2B), analyzing their performance before and after fine-tuning. The results demonstrate that VLMs, particularly Qwen2.5-VL-3B-Instruct, significantly reduce false positives and support fuzzy reasoning in fire-related scenes. This study affirms the potential of VLM-based multimodal reasoning as a robust solution for intelligent, context-aware fire detection.

For edge deployment, a promising direction is to replace free-form caption generation with a compact vision-language classifier optimized using quantization and parameter-efficient fine-tuning. This design avoids the high latency of generative decoding while preserving the contextual reasoning benefits of multimodal input, making it more suitable for real-time fire recognition on resource-limited devices.

For future work, we plan to expand our dataset with more diverse fire scenes, including synthetic and infrared images, to enhance the model’s robustness. We will also explore video-based models, such as 3D CNNs or Transformers, to gain a deeper understanding of how fire changes over time. Additionally, integrating visual data with other sensor modalities, such as thermal, gas detection, and sound sensors, can accurately detect fire events and reduce the likelihood of false alarms.

## Data Availability

The datasets generated and/or analysed during the current study are available in the Github or Kaggle repository: - https://github.com/tmdeptrai/fire-context-aware-dataset - https://www.kaggle.com/datasets/duongtm3012/confire.

## Code availability

The source code is available at https://github.com/tmdeptrai/ContextFire-VLM/.
